# Deciphering the pathogenic role of a variant with uncertain significance for short QT and Brugada syndromes using gene‐edited human‐induced pluripotent stem cell‐derived cardiomyocytes and preclinical drug screening

**DOI:** 10.1002/ctm2.646

**Published:** 2021-12-26

**Authors:** Ibrahim El‐Battrawy, Huan Lan, Lukas Cyganek, Lasse Maywald, Rujia Zhong, Feng Zhang, Qiang Xu, Jihyun Lee, Eliane Duperrex, Andreas Hierlemann, Ardan M. Saguner, Firat Duru, Boldizsar Kovacs, Mengying Huang, Zhenxing Liao, Sebastian Albers, Jonas Müller, Hendrik Dinkel, Lena Rose, Alyssa Hohn, Zhen Yang, Lin Qiao, Yingrui Li, Siegfried Lang, Mandy Kleinsorge, Andreas Mügge, Assem Aweimer, Xuehui Fan, Sebastian Diecke, Ibrahim Akin, Guang Li, Xiaobo Zhou

**Affiliations:** ^1^ First Department of Medicine Faculty of Medicine University Medical Centre Mannheim (UMM), University of Heidelberg Mannheim Germany; ^2^ Key Laboratory of Medical Electrophysiology of Ministry of Education and Medical Electrophysiological Key Laboratory of Sichuan Province Institute of Cardiovascular Research, Southwest Medical University Luzhou China; ^3^ Stem Cell Unit, Clinic for Cardiology and Pneumology University Medical Center Göttingen Göttingen Germany; ^4^ Department of Biosystems Science and Engineering Bioengineering Laboratory Basel Switzerland; ^5^ Department of Cardiology Electrophysiology Division University Heart Center Zurich Zurich Switzerland; ^6^ DZHK (German Center for Cardiovascular Research) Partner Site Heidelberg‐Mannheim and Göttingen Mannheim Germany; ^7^ Department of Cardiology and Angiology Bergmannsheil Bochum, Medical Clinic II Ruhr University Bochum Germany; ^8^ Max Delbrück Center for Molecular Medicine Berlin Germany

**Keywords:** brugada syndrome, cardiac death, channelopathy, short QT syndrome

Dear Editor,

In the present study, we show that the Calcium Voltage‐Gated Channel Auxiliary Subunit Beta 2 (CACNB2) variant c.1439C>T/p.S480L is linked to the clinical phenotype of short QT syndrome 5 (SQTS5) overlapped with Brugada syndrome (BrS). The PI3K pathway may contribute to the arrhythmogenesis of the disease. PI3K‐activator and amiodarone but not sotalol may be effective drugs for treating arrhythmias in SQTS5‐patients carrying but not limited to this variant.

The SQTS is characterized by a shortening of the corrected QT (QTc) interval, which has been linked to sudden cardiac death.^1^, ^2^ Implantable cardioverter‐defibrillator therapy is associated with numerous complications.^3^ Therefore, drug therapy is important to optimize the treatment of SQTS patients. Recently published data have reported that hydroquinidine is effective in prolonging the QTc interval in SQTS patients. Notably, data have shown that hydroquinidine might exert different effects depending on the genetic variant and/or SQTS form.^4^, ^5^

Based on the limited evidence of the clear role of variants in calcium channel subunits in SQTS and the absence of alternative therapies in this rare cohort,^6^ we aimed to use cardiomyocytes from induced pluripotent stem cells (hiPSC‐CMs) derived from a SQTS5‐patient overlapped with BrS carrying a variant in CACNB2 to study the significance of the variant for the clinical phenotype by combining gene editing and electrophysiological analysis in order to identify possible effective drugs for the disease. HiPSC‐CMs offered advantages over other models to model channelopathies in the dish.^7^, ^8^

For this study, human iPSC lines from one SQTS patient, from two healthy donors, and two Clustered Regularly Interspaced Short Palindromic Repeats (CRISPR)/Cas9 gene‐edited hiPSC lines were used (Figure S1A,B). The hiPSC lines were verified for pluripotency (Figure S1C–E) and were differentiated into cardiomyocytes (Figure S1F), showing cardiac action potential (AP) features (Figure S2).

The SQTS patient showed an abbreviated QTc interval with a QTc of 330 ms (Figure 1A) and was admitted due to an aborted cardiac arrest. A genetic screening of SQTS related genes of this patient detected a variant, namely c.1439C>T/p.S480L (dbSNP rs121917812; Clinvar RCV000010155.3; NM_000724.4: c.1439C>T; NM_201590.3: c.1442C>T) in CACNB2, a beta‐subunit of L‐type calcium channel (Figure 1C). Sequencing of SQTS related genes confirmed the existence of the same variant in other first or second‐degree relatives (Figure 1B,C) with a SQTS and BrS phenotype.

**FIGURE 1:**
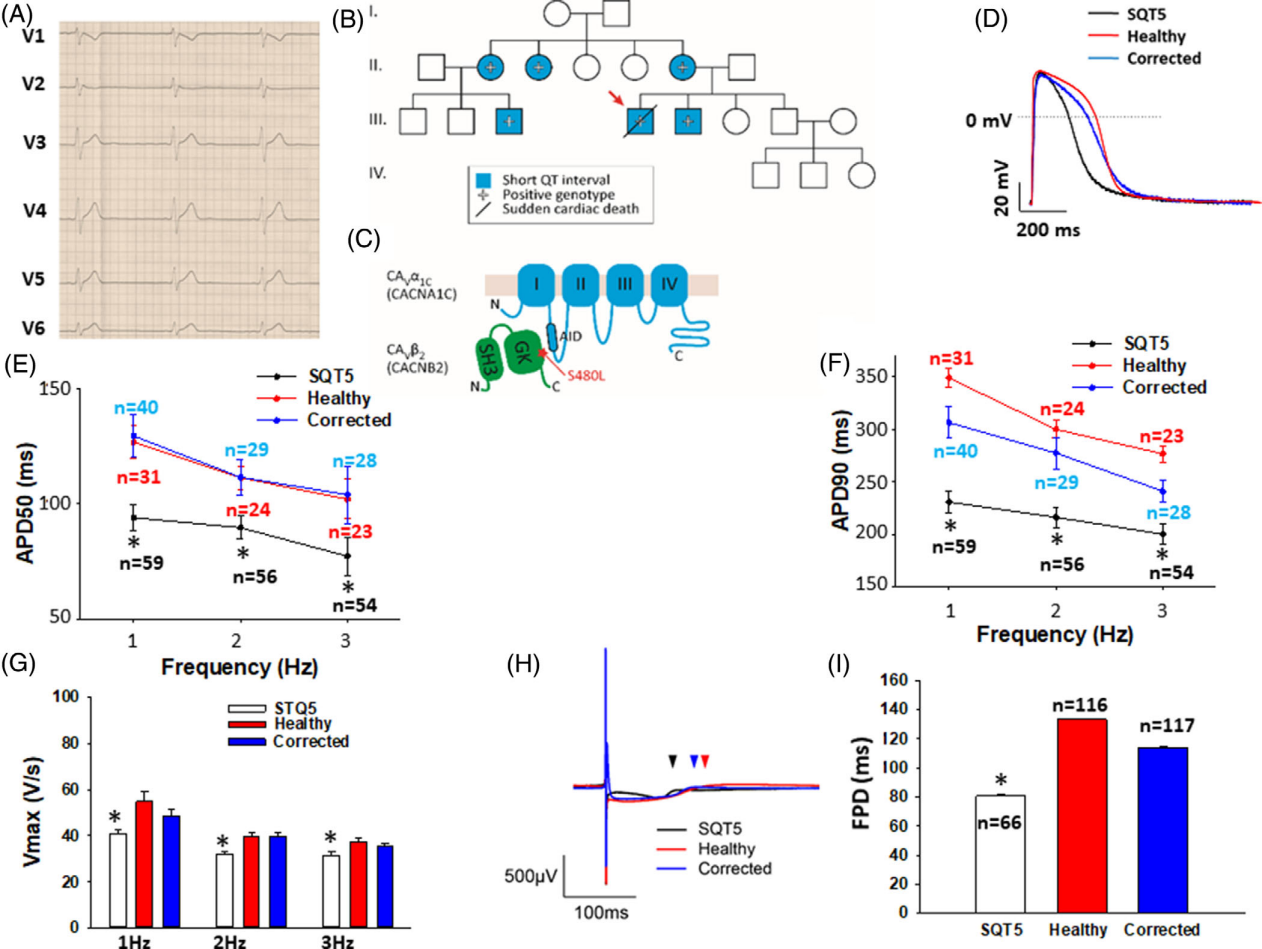
Clinical and biological characteristics of the short QT syndrome 5 (SQTS5) patient. (A) electrocardiogram (ECG) presenting corrected QT (QTc) abbreviation (QTc = 330 ms, QT 330 ms at heart rate 60 beats per min). (B) Family pedigree of the SQTS5‐patient. The patient recruited for this study is indicated by the arrow. (C) Scheme of the CaVα1C‐ and CaVβ2‐subunits showing that the α‐interacting domain (AID) binds to the β‐subunit guanylate kinase domain (GK). The variant S480L in CaVβ2 might interfere proper interaction of the subunits. (D–G) Action potentials paced at 1 to 3 Hz were recorded by patch‐clamp techniques and the duration at 50% (APD50) and 90% (APD90), as well as the maximal velocity of depolarization (V_max_), were compared among cardiomyocytes from induced pluripotent stem cells (hiPSC‐CMs) from the SQTS5‐patient (SQTS5), the healthy donor (Healthy) and CRISPR‐corrected SQTS5 (Corrected) cells. (D) Representative action potential (AP) traces of cells from each cell line at 1 Hz. (E) Averaged values of APD50 in each cell line at 1–3 Hz. (F) Averaged values of APD90 in each cell line at 1–3 Hz. (G) Averaged values of V_max_ in each cell line at 1–3 Hz. (H) Representative traces of field potentials in cells of each cell line. (I) Averaged values of field potential duration (FPD) in each cell line. “*n*” numbers represent the number of cells. *p < .05 versus Healthy according to the analysis of one‐way ANOVA with Holm‐Sidak post‐test.

The durations of AP at 50% (APD50) and 90% repolarization (APD90) were significantly shorter in SQTS5 cells compared to that in the cells from the healthy donor and isogenic control cells (Figure 1D–F). The maximal depolarization speed (V_max_) of APs was decreased (Figure 1G). Of note, the APD‐shortening remained at all the tested frequencies. In addition, HD‐MEA recordings detected that the field potential duration in SQTS5‐hiPSC‐CMs was also significantly shorter compared to the healthy or isogenic control (Figure 1H–I). In recordings of spontaneous APs, the frequency of cell beating in SQTS5‐hiPSC‐CMs was significantly slower than that in healthy cells, without difference in maximal diastolic potential (Figure S3C,D,G,H). After the APD parameters were corrected for beating frequency (Bazett´s correction), the corrected APDs were also shorter in SQTS5‐hiPSC‐CMs (Figure S3C–F).

The L‐type calcium channel current (I_Ca‐L_) was significantly decreased in SQTS5‐hiPSC‐CMs as compared to that from the healthy control and isogenic control (Figure 2A,B). The activation curve of I_Ca‐L_ in SQTS5‐cells was shifted to more positive potentials compared with that in isogenic control hiPSC‐CMs. Besides, the inactivation curve shifted to more negative potentials, and recovery from inactivation decelerated in SQTS5‐hiPSC‐CMs compared with both healthy and isogenic control cells (Figure 2C–H). Western blot and immunostaining analyses detected that the protein expression level of CACNB2 was significantly decreased in SQTS5‐hiPSC‐CMs (Figure S4). The alpha subunit of L‐type Ca^2+^ channel CACNA1C was slightly increased in cell lysates (Figure S4) but not in the cell membrane (Figure S4).

**FIGURE 2:**
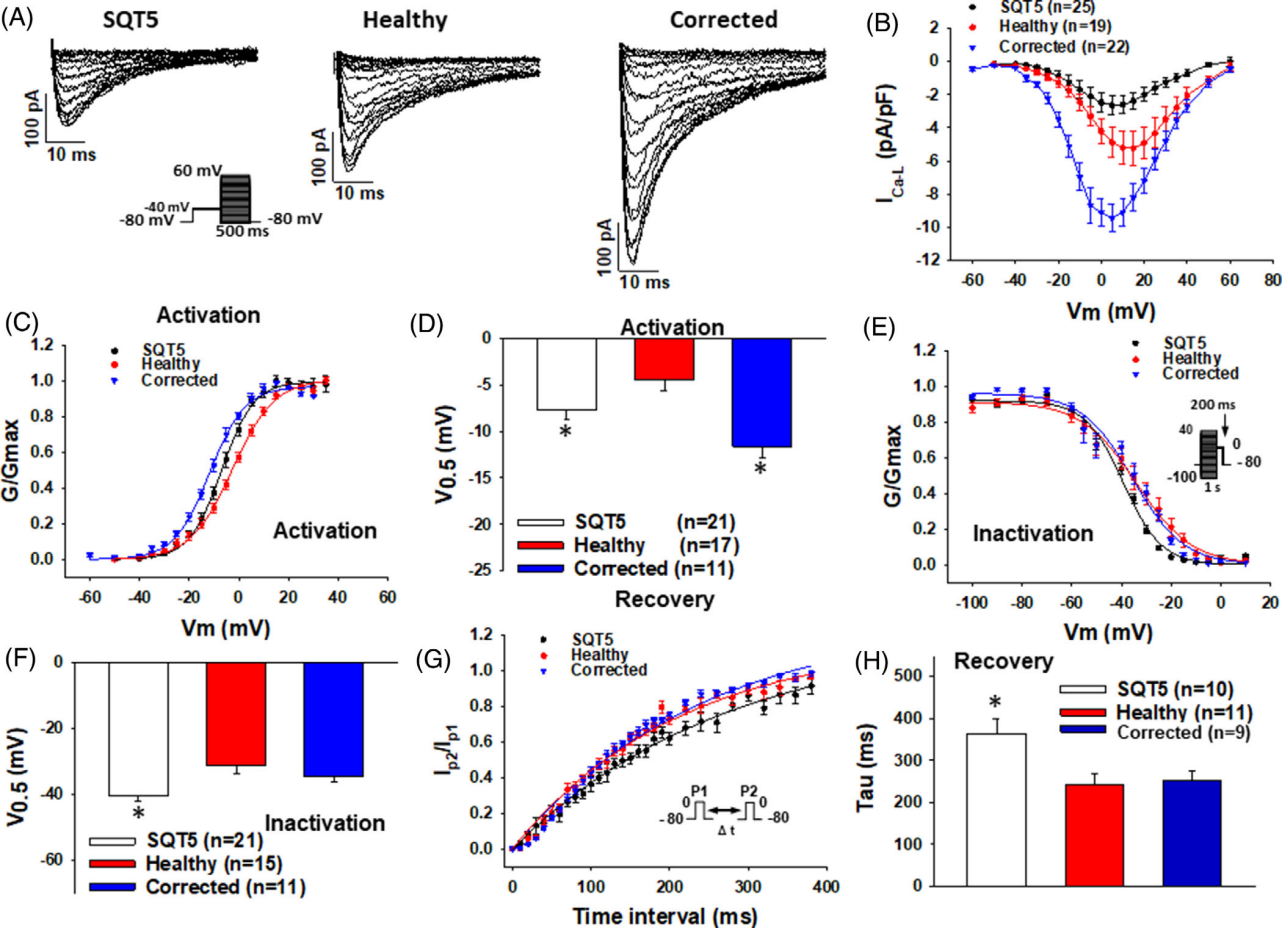
L‐type calcium channel current decreased in SQTS5‐hiPSC‐CMs. For analyzing the current amplitude and activation of L‐type calcium channels, the protocol indicated in (A) (inset) was used. For analyzing inactivation and recovery from inactivation of L‐type calcium channels, protocols indicated in (E) (inset) and (G) (inset) were used, respectively. (A) Representative current traces in cardiomyocytes from induced pluripotent stem cells (hiPSC‐CMs) from the patient (short QT syndrome 5 [SQTS5]), the healthy donor (Healthy), and the CRISPR‐corrected (Corrected) cells. (B) The current‐voltage (I‐V) relationship curves of L‐type calcium channel currents (I_Ca‐L_) in cells from each group. (C) The activation curves of I_Ca‐L_ in cells from each group. (D) The half‐maximum activation potential (V_0.5_) of I_Ca‐L_ in cells from each group. (E) The inactivation curves of I_Ca‐L_ in cells from each group. (F) The half‐maximum inactivation potential (V0.5) of I_Ca‐L_ in cells from each group. (G) The curves of recovery from inactivation of I_Ca‐L_ in cells from each group. (H) The time constants (τ) of recovery from inactivation of I_Ca‐L_ in cells from each group. “*n*” numbers represent the number of cells. *p < .05 versus Healthy according to the analysis of one way analysis of variance (ANOVA) with Holm‐Sidak post‐test

Since loss‐of‐function of the sodium channel is a main feature of BrS, the peak sodium channel current (I_Na_) and their kinetics were assessed (Figure 3A,B and Figure S5A–F). In SQTS5‐hiPSC‐CMs, the peak I_Na_ and activation were significantly suppressed compared to healthy cells.

**FIGURE 3:**
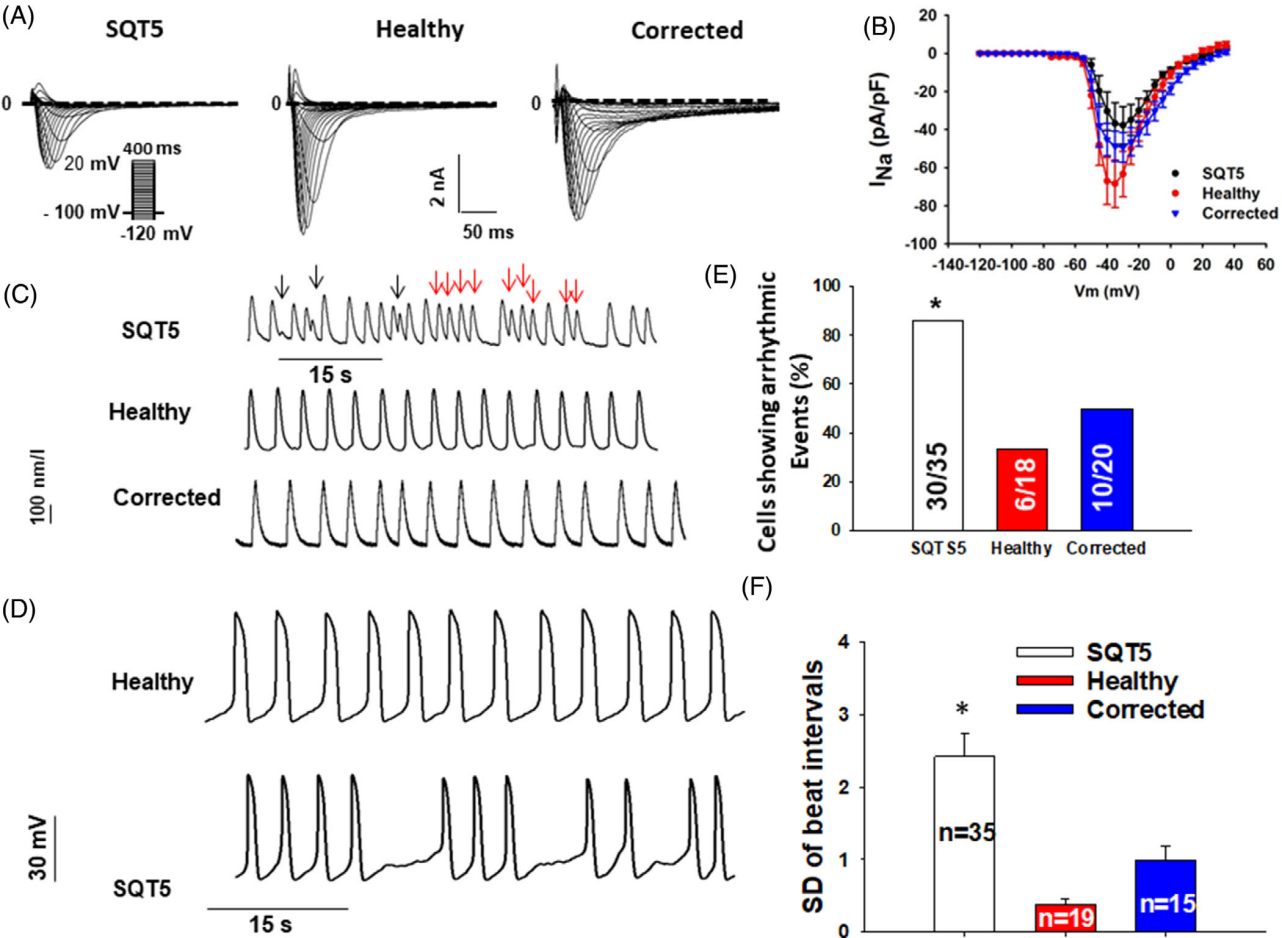
Peak sodium channel current decreased in SQTS5‐hiPSC‐CMs and arrhythmic events were increased. For analyzing the current amplitude and activation of sodium channels, the protocol indicated in (A) (inset) was used. For analyzing inactivation and recovery from inactivation, protocols indicated in S5C (inset) and S5E (inset) were used, respectively. (A) Representative current traces in cardiomyocytes from induced pluripotent stem cells (hiPSC‐CMs) from the patient (short QT syndrome 5 [SQTS5]), the healthy donor (Healthy), and the CRISPR‐corrected (Corrected) cells. (B) The current‐voltage (I‐V) relationship curves of sodium channel currents (I_Na_) in cells from each group. Spontaneous calcium transients and action potentials were recorded in spontaneously beating hiPSC‐CMs from the patient (SQTS5), the healthy donor (Healthy), and the CRISPR‐corrected cells (Corrected). The occurrence of arrhythmic events (irregular or triggered beats or EAD‐like events) was compared among the three cell groups. (C) Representative traces of calcium transients in cells from each line. Arrhythmic events are marked by arrows (black, EAD‐like events; red, triggered beats). (D) Representative traces of spontaneous action potentials in cells from the healthy donor (Healthy) and the patient (SQTS5), showing trigeminus‐like arrhythmic events in SQTS5‐hiPSC‐CMs. (E) Percentage of cells showing arrhythmic events. The numbers given represent the number of cells. *p < .05 versus Healthy according to the Fisher‐test. (F) The standard deviation of cell beat intervals in SQTS5‐hiPSC‐CMs, healthy donor and isogenic control cells. “*n*” numbers represent the number of cells. *p < .05 versus Healthy according to the analysis of one way analysis of variance (ANOVA) with Holm‐Sidak post‐test

To test the arrhythmogenicity of hiPSC‐CMs of the SQTS5‐patient, spontaneous calcium transients and spontaneous APs were recorded. Compared to healthy donor and isogenic control cells, a higher number of SQTS5‐cells displayed arrhythmic events (irregular or triggered beats) (Figure 3C–F). The interval variability (standard deviation of beating intervals) in SQTS5‐hiPSC‐CMs was larger than that in donor and isogenic control cells (Figure 3C–F).

For details about methods, please see Supporting Information. As expected, insertion of the present CACNB2 variant using CRISPR/Cas 9 in a further control cell line (Figure S6) led to a loss‐of‐function of I_Ca‐L_ and an APD‐shortening with a reduction of V_max_ (Figure S7).

Quinidine and amiodarone prolonged APD (Figures S8 and S9) but only amiodarone showed a significant antiarrhythmic effect (Figure S9). Sotalol showed no effect in SQTS5‐cells although it prolonged APD in healthy cells (Figures S10 and S11).

Since the PI3K pathway may influence ion channel function and QT interval,^9^, ^10^ we measured the protein levels of PI3K and Akt. Both total and phosphorylation levels of Akt and PI3K were decreased in SQTS5‐hiPSC‐CMs compared to healthy cardiomyocytes (Figure 4A–C). Whereas a PI3K‐activator (IGF‐1, 100 ng/ml) reduced the interval variability and arrhythmic events, a PI3K blocker (alpelisib, 5 μM) enhanced both (Figure 4D–F). In addition, the PI3K‐activator prolonged the APD (Figure 4G,H).

**FIGURE 4:**
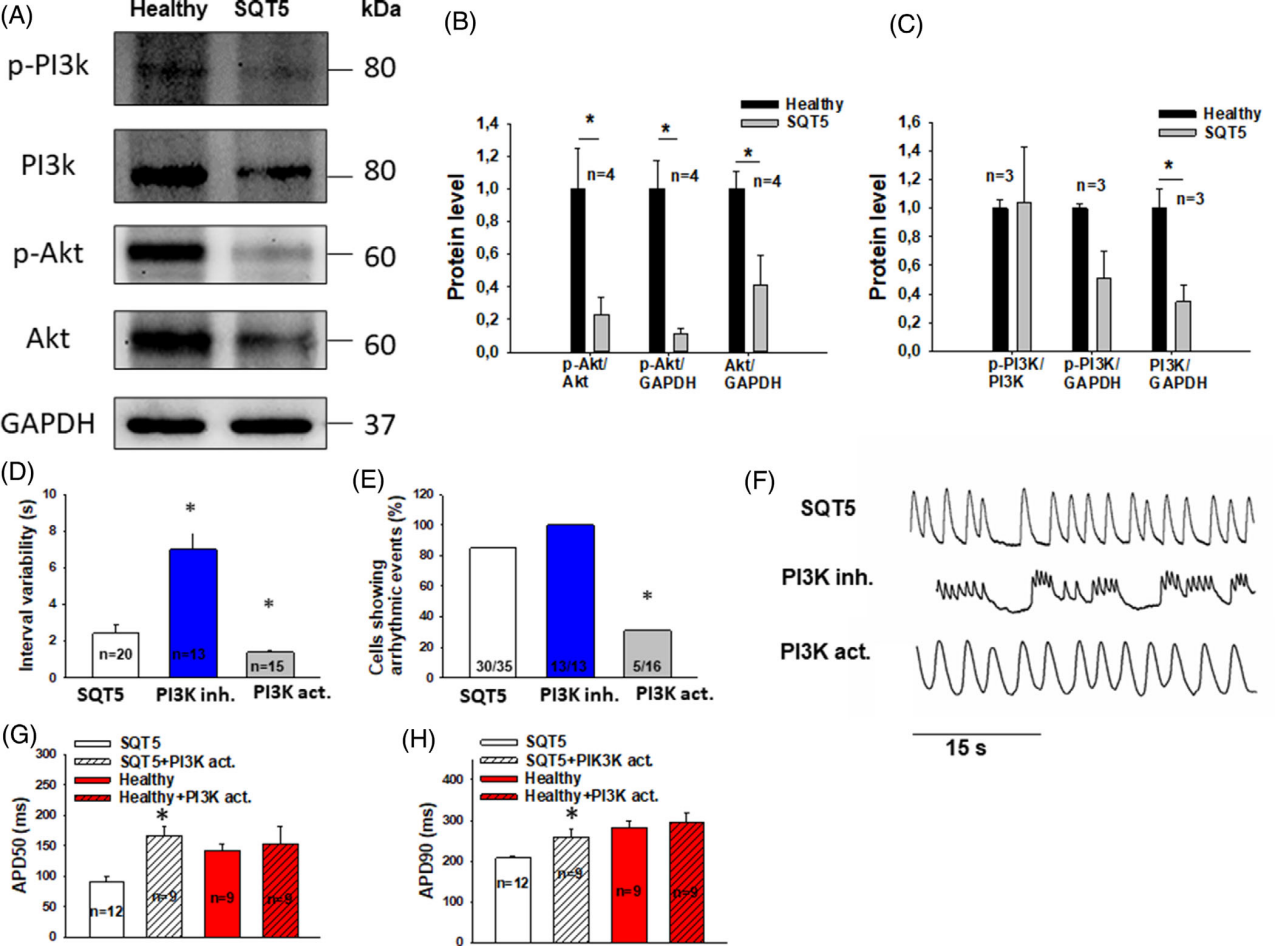
PI3 signaling was altered in SQTS5‐hiPSC‐CMs. (A) Representative bands of western blots showing the expression levels of phosphorylated Akt (p‐Akt) and PI3K (p‐PI3K) or total Akt (Akt) and PI3K (PI3K). (B) Mean values of phosphorylated and total Akt protein levels normalized to that of healthy cells. (C) Mean values of phosphorylated and total PI3K protein levels normalized to that of healthy cells. “*n*” represents the number of experiments. (D) Interval variability in the absence (Ctr) and presence of a PI3K inhibitor alpelisib (PI3K inh.) or PI3K activator IGF‐1 (PI3K act.). (E) Percentage of cells showing arrhythmic events in the absence (Ctr) and presence of alpelisib (PI3K inh.) or IGF‐1 (PI3K act.). “*n*” represents the number of cells. (F) Representative traces of calcium transients in an SQTS5‐hiPSC‐CM in the absence (short QT syndrome 5 [SQTS5]) and presence of alpelisib (PI3K inh.) or IGF‐1 (PI3K act.). (G) Mean values of action potential duration at 50% repolarization (APD50) in the absence (SQTS5, Healthy) and presence of IGF‐1 (SQTS5 + PI3K act. and Healthy + PI3K act.) (H) Mean values of action potential duration at 90% repolarization (APD90) in the absence (SQTS5, Healthy) and presence of IGF‐1 (SQTS5 + PI3K act. and Healthy + PI3K act.). “*n*” represents the number of cells. *p < .05 versus SQTS5 according to one‐way analysis of variance (ANOVA) with Holm‐Sidak post‐test

In conclusion, the variant c.1439C>T/p.S480L in the CACNB2 gene is responsible for phenotypic changes of SQTS5 overlapping with BrS. Amiodarone reduces arrhythmic events in the dish and based on this, we may assume that it may be clinically more effective than quinidine for treating SQTS5 and /or BrS. The PI3K/Akt activity can be reduced in SQTS5 and an activator of this signaling pathway may rescue the phenotype.

## Funding

This study was supported by the Hector‐Stiftung and DZHK (German Center for Cardiovascular Research. Furthermore this study is supported by NSFC 81870261, Sichuan Youth Science and Technology Innovation Research Team 2020JDTD0024, Collaborative Innovation Center for the Prevention and Treatment of Cardiovascular Diseases in Sichuan Province xtcx2016‐14 and interrnational cooperation project of Science & Technology Department of Sichuan Province (No. 2020YFH0139)

## Supporting information



Supporting Information

Supporting Information‐Supplementary figure 1

Supporting Information‐Supplementary figure 2

Supporting Information‐Supplementary figure 3

Supporting Information‐Supplementary figure 4

Supporting Information‐Supplementary figure 5

Supporting Information‐Supplementary figure 6

Supporting Information‐Supplementary figure 7

Supporting Information‐Supplementary figure 8

Supporting Information‐Supplementary figure 9

Supporting Information‐Supplementary figure 10

Supporting Information‐Supplementary figure 11
