# Supplementary material for: Deciphering the pathogenic role of a variant with uncertain significance for short QT and Brugada syndromes using gene‐edited human‐induced pluripotent stem cell‐derived cardiomyocytes and preclinical drug screening
Source: Clin Transl Med. 2021 Dec 26;11(12):e646. doi: 10.1002/ctm2.646 (PMC8710296; doi:10.1002/ctm2.646)
Supplement: Supplementary file 1 — Supporting Information [file CTM2-11-e646-s006.DOC]

**Supplementary information**

# Deciphering the pathogenic role of a variant with uncertain significance for short QT and Brugada syndrome using gene-edited human-induced pluripotent stem cell-derived cardiomyocytes and preclinical drug screening

**Running title: Short QT and Brugada Syndrome with CACNB2 variant**

Ibrahim El-Battrawy^1,6,7^*****, Huan Lan^2^*, Lukas Cyganek^3,6*^, Lasse Maywald^1,6*^, Rujia Zhong^1^, Feng Zhang^1^, Qiang Xu^1^, Jihyun Lee^4^, Eliane Duperrex^4^, Andreas Hierlemann^4^, Ardan M. Saguner^5^, Firat Duru^5^, Boldizsar Kovacs^5^, Mengying Huang^1^, Zhenxing Liao^1^, Sebastian Albers^1,5^, Jonas Müller^1,5^, Hendrik Dinkel^1,6^, Lena Rose^1^, Alyssa Hohn^1^, Zhen Yan^1^, Lin Qiao^1^, Yingrui Li^1^, Siegfried Lang^1,6^, Mandy Kleinsorge^3,5^, Andreas Mügge^7^, Assem Aweimer^7^, Xuehui Fan^1^, Martin Borggrefe^1,6^, Sebastian Diecke^8┼^, Ibrahim Akin^1,5┼^, Guang Li^2#┼^ and Xiaobo Zhou^1,5,2#┼^

^1^First Department of Medicine, Faculty of Medicine, University Medical Centre Mannheim (UMM), University of Heidelberg, Mannheim, Germany

^2^Key Laboratory of Medical Electrophysiology of Ministry of Education and Medical Electrophysiological Key Laboratory of Sichuan Province**,** Institute of Cardiovascular Research, Southwest Medical University, Luzhou, Sichuan, China

^3^Stem Cell Unit, Clinic for Cardiology and Pneumology, University Medical Center Göttingen, Göttingen, Germany

^4^Bioengineering Laboratory, Department of Biosystems Science and Engineering, ETH Zürich, Mattenstrasse 26, Basel, 4058, Switzerland.

^5^Electrophysiology Division, Department of Cardiology, University Heart Center Zurich, Raemistrasse 100, 8091 Zurich, Switzerland

^6^DZHK (German Center for Cardiovascular Research), Partner Site, Heidelberg-Mannheim and Göttingen, Mannheim, Germany

^7^Bergmannsheil Bochum, Medical Clinic II, Department of Cardiology and Angiology, Ruhr University, Bochum, Germany

^8^Max Delbrück Center for Molecular Medicine, Berlin, Germany

*Equally contributed

# Corresponding authors.

┼Drs. Zhou, Li, Akin and Diecke share senior authorship.

**Address for correspondence**:

1. Xiaobo Zhou, MD. First Department of Medicine, University Medical Centre Mannheim, Theodor-Kutzer-Ufer 1-3, 68167 Mannheim, Germany. Phone: 0049-621-383-1448. Fax: 0049-621-383-1474. E-mail: xiaobo.zhou@medma.uni-heidelberg.de
2. Guang Li, PhD. Key Laboratory of Medical Electrophysiology of Ministry of Education and Medical Electrophysiological Key Laboratory of Sichuan Province**,** Institute of Cardiovascular Research, Southwest Medical University, 646000 Luzhou, Sichuan, China. Phone: 008613882776203. Fax:0830-3161222. E-mail: liguang@swmu.edu.cn

**Generation of human iPS cells**

The hiPSC lines isSQTSb1.4 (UMGi129-A.4) and isSQTSb1.5 (UMGi129-A.5) were generated from primary human fibroblasts derived from a skin biopsy of the patient in feeder free culture conditions using the integration-free CytoTune-iPS 2.0 Sendai Reprogramming Kit (Thermo Fisher Scientific, #A16517) with the reprogramming factors OCT4, KLF4, SOX2, c-MYC according to manufacturer’s instructions with modifications. The generated hiPSCs were characterized for their pluripotency, as described previously^1^. The human iPSC lines from healthy donors ipWT1.3/UMGi014-B.3 (provided by Stem Cell Unit, Göttingen, Germany) and LHpb-YaabC3 (provided by Zhongyu Medical Biotech, Guangzhou, China) were generated from dermal fibroblasts and urine-derived cells, respectively, using integration-free episomal reprogramming plasmids and described previously. The hiPSC lines were maintained on Matrigel-coated (growth factor reduced, BD Biosciences) plates, passaged every 4-6 days with EDTA dissociation medium and cultured in StemMACS iPS-7 Brew XF medium (Miltenyi Biotech) supplemented with 2 μM Thiazovivin (Merck Millipore) for 24 h after passaging with daily medium change. Directed differentiation of hiPSCs into hiPSC-CMs was performed via WNT signaling modulation and subsequent metabolic selection, as previously described. A minimum of three individual differentiation experiments of the individual iPSC lines were used for each analysis if not indicated otherwise. Cell cultures were incubated in a humidified incubator with 5% CO_2_ and 21% O_2_ at 37°C.

**CRISPR/Cas9 genome editing of human iPS cells**

Genetic correction of the patient line isSQTSb1.4 (UMGi129-A.4) was performed using ribonucleoprotein-based CRISPR/Cas9 by targeting exon 20 of the CACNB2 gene. The guideRNA target sequence, with PAM in bold, was: 5’- CACTGCGATGGTTGTGGTGT**GGG**-3’. For homology-directed repair, a single-stranded oligonucleotide with 45 bp homology arms including the wild type sequence was used. Individual iPSC clones were expanded for approximately 2-3 weeks and analyzed for genetic modification by Sanger sequencing. Two CRISPR-corrected iPSC lines isSQTSb1-corr.A (UMGi129-A-1.A) and isSQTSb1-corr.D (UMGi129-A-1.D) were used for analysis. Introduction of the CACNB2 S480L variant in control iPSC line LHpb-YaabC3 was performed by plasmid-based CRISPR/Cas9 using the pSpCas9(BB)-2A-GFP plasmid (Addgene) with the respective guideRNA sequence and a single-stranded oligonucleotide containing a single base-pair substitution and two silent SNPs to introduce Kpn1 restriction site and to prevent repeat cutting of genomic DNA. The guideRNA target sequence, with PAM in bold, was: 5’- CGCGACTTGTCCCACTGCGA**TGG**-3’. Two positive iPSC clones with introduced SNP were expanded for further experiments.

**Immunostaining**

The cells in chamber were washed by phosphate-buffered saline (PBS) and fixed by 4% paraformaldehyde for 10 minutes, blocked in blocking solution (5% fetal bovine serum, 0.5% triton in PBS) for 30 minutes. Then the cells were incubated overnight at 4 ˚C with monoclonal anti-α-actinin (Sarcomeric) (A7811, 1:400; Sigma-Aldrich, Merck KGaA, Darmstadt, Germany); anti-Cardiac Troponin T antibody [1C11] (ab8295, 1:400; Abcam, Cambridge, UK); anti-Myl2 antibody (NBP1-30249,1:400; Novus, Wiesbaden Nordenstadt, Germany). The second antibody for immunofluorescence staining was anti-rabbit/mouse IgG (H+L), F(ab')2 Fragment (1:2000; Cell Signaling, USA). Pictures were captured by the microscope (Olympus IX, Tokyo, Japan) equipped with a DP70 CCD digital camera (Olympus, Tokyo, Japan). Three randomly fields per slice were captured, and plaque area was calculated by Image-Pro Plus software (Mediacybernetics, Acton, MA, USA).

**Western blotting**

The cells were collected by PBS. The lysate was centrifuged at 12,000 × g, 4 ˚C for 30 minutes. The supernatant was used for western blotting. BCA Protein Assay kit (23227, Thermo, Rockford, USA) was used to measure the total protein concentration. The following primary antibodies, including Glyceraldehyde 3-phosphate dehydrogenase (GAPDH) (14C10) antibody (2118S, 1:10000; Cell Signaling, Chicago, IL, USA); anti-Ca_v_1.2 (CACNA1C) antibody (ASC003, 1:1000; Alomone Labs, Israel); anti-CACNB2 antibody (ab54920,1:1000; Abcam, Cambridge, UK ); anti-PI3K antibody (4257T, 1:1000; Cell Signaling Technology, USA); anti-phospho-PI3K antibody (4228T, 1:1000; Cell Signaling Technology, USA); anti-Akt antibody (9272S, 1:1000; Cell Signaling Technology, USA); anti-phospho-Akt antibody (4060T, 1:1000; Cell Signaling Technology, USA). The second antibody was anti-rabbit IgG-peroxidase produced in goat (A0545, 1:2000; Sigma-Aldrich,Merck KGaA, Darmstadt, Germany) or anti-mouse IgG-peroxidase produced in goat (A3682, 1:2000; Sigma-Aldrich,Merck KGaA, Darmstadt, Germany). The target protein bands were analyzed by Image-Pro Plus software (Mediacybernetics, Acton, MA, USA).

**Patch-clamp**

Standard patch-clamp recording techniques were used to measure the action potential (AP) and channel currents in the whole-cell configuration at room temperature. Patch electrodes were pulled from borosilicate glass capillaries (MTW 150F; world Precision Instruments, Inc., Sarasota, FL) using a DMZ-Universal Puller (Zeitz-Instrumente-Vertriebs GmbH, Martinsried, Germany) and filled with pre-filtered pipette solution (see below). Pipette resistance ranged from 1-2 MΩ and 4-5 MΩ for current and AP measurements, respectively. Electrode offset potentials were zero-adjusted before a Giga-seal was formed. After a Giga-seal was obtained, fast capacitance was first compensated and then the membrane under the pipette tip was disrupted by negative pressure to establish the whole-cell configuration. Signals were acquired at 10 kHz and filtered at 2 kHz with the Axon 200B amplifier and Digidata 1440A digitizer hardware as well as pClamp10.2 software (Molecular Devices, Sunnyvale, CA). Spontaneous and paced APs were recorded in current clamp mode. For recording APs paced at fixed frequencies, brief current pulses (2 ms, 1 nA) were applied with different frequencies to trigger APs.

The bath solution (PSS) for AP measurements contained (mmol/l): 130 NaCl, 5.9 KCl, 2.4 CaCl_2_, 1.2 MgCl_2_, 11 glucose, 10 HEPES, pH 7.4 (NaOH). The pipette solution contains (mmol/l): 10 HEPES, 126 KCl, 6 NaCl, 1.2 MgCl_2_, 5 EGTA, 11 glucose and 1 MgATP, pH 7.2 (KOH).

The bath solution for L-type (I_Ca-L_) calcium channel current recordings contained (mmol/l): 140 TEA-Cl, 5 CaCl_2_, 1 MgCl_2_, 10 HEPES, 1 µM E-4031, 0.02 TTX, 3 4-AP, pH 7.4 (CsOH). Microelectrodes were filled with (mmol/l): 6 NaCl, 135 CsCl, 2 CaCl_2_, 3 MgATP, 2 TEA-Cl, 5 EGTA, 10 HEPES, pH7.2 (CsOH).

**Electrophysiological measurements using HD-MEAs**

Since several years, microelectrode arrays (MEAs) have been used as a platform to characterize electrophysiological phenotypes of iPSC-derived cardiomyocytes from patients with hereditary cardiac diseases, such as the long QT interval ^2, 3^ for assessing drugs in vitro ^4^. Cardiac field potentials are measured non-invasively, whiles the intact cell membrane is located close to the electrode surface. We measured field potentials of cardiomyocytes using high-density MEAs (HD-MEAs) ^5^ which accommodate 26,400 electrodes in an area of ~4x2 mm^2^. The electrodes are sized 5x9um^2^ and feature a porous Pt-black surface coating, which enables low-impedance measurements of field potentials. After seeding cardiomyocytes on the HD-MEA, the cells were allowed to recover for >7 days until they started to beat spontaneously. Field potentials were measured in a humidified incubator with 5% CO_2_.

**Measurement of intracellular calcium transients**

To measure the intracellular Ca^2+^ transients, cells were loaded with the fluorescent Ca^2+^-indicator Fluo-3 AM. First, 1.5 ml PSS (see below) was added into a petri dish with hiPSC-CMs cultured for 2 to 4 days. Then, 50 µg of the membrane permeable acetoxymethyl ester derivative of Fluo-3 was dissolved in 44 µl of the Pluronic F-127 stock solution (20% w/v in DMSO) to get a 1 mM Fluo-3 AM stock solution, which can be stored at -20 °C for a maximum of 1 week. Next, 15 µl of the Fluo-3 AM stock solution were added into 1.5 ml PSS resulting in a final concentration of 10 µM Fluo-3 and the dish was agitated carefully. The cells were incubated at room temperature for 10 minutes in an optically opaque box to protect from light. Thereafter, the PSS was carefully sucked out and discarded and the cells were washed with PSS for 4-5 times. Finally, the cells in PSS were kept at room temperature for about 30 minutes for de-esterification before measurements. After de-esterification, the fluorescence of the cells was measured by using Cairn Optoscan calcium imaging system (Cairn Research, UK). Fluorescence is excited by 488 nm and emitted at 520 nm and spontaneous Ca^2+^ transients were recorded. Arrhythmic events are defined as the presence of early afterdepolarization (EAD)-like events.

**Drugs**

Drugs were applied to a cell sequentially from low to high concentrations by a perfusion pipette. The tested concentrations were selected according to previous or our preliminary studies in hiPSC-CMs. Quinidine, amiodarone, and sotalol are from Sigma-Aldrich, 4-AP from RBI, TTX from Carl Roth.

**Statistical analysis**

Data are shown as mean ± SEM and were analyzed using InStat© (GraphPad, San Diego, USA) and SigmaPlot 11.0 (Systat GmbH, Germany). For data of more than two groups multiple comparisons with one-way ANOVA and Holm-Sidak post-test were performed. Repeated measures ANOVA with Bonferroni correction was used for repeated measurements in same cells. Paired t-test was used for comparisons of data before and after application of a drug. To compare categorical variables, the Fisher-test was used. p<0.05 (two-tailed) was considered significant.

**Ethics statement**

For this study, human iPSC lines from one SQTS patient, from two healthy donors and two CRISPR/Cas9 gene-edited hiPSC lines were used. Skin biopsies were obtained with written informed consent. The application of human iPS cells for this study has been approved by the Ethics Committee of the Medical Faculty Mannheim, University of Heidelberg (approval number: 2009-350N-MA), the Ethics Committee of University Medical Center Göttingen (approval number: 10/9/15) and the Ethics Committee of Southwest Medical University (approval number: XNYD2018007). The study was carried out in accordance with the approved guidelines and conducted in accordance with the Helsinki Declaration revised in 2013 (<https://www.wma.net/what-we-do/medical-ethics/declaration-of-helsinki/>). For experiments, cells from at least 5 differentiations were obtained.

**Results**

**Clinical data**

At age of 25, the patient was admitted to the hospital with aborted SCD and short QT interval (**figure 1 A**). An ICD was implanted for secondary prophylaxis. In addition, the patient was also treated with quinidine. During quinidine treatment, no ventricular arrhythmia but atrial flutter appeared. A cascade family screening including physical examination, serial ECGs, 24-hour Holter monitoring, echocardiogram, exercise testing, and cardiac MRI confirmed familiar SQTS. Other Genes except CACNB2 were screened including CACNA1C, KCNH2, KCNJ2, KCNQ1 and SCN5A, however no genetic variation was found. The detected variant in CACNB2 gene (**figure S1 B**), which encodes a regulatory subunit of L-type calcium channel (**figure 1C**), is classified as pathogenic for Brugada syndrome type 4 and as VUS for SQTS (SQT5)^6^. Sequencing of SQTS related genes confirmed the existence of the same variant in other first or second degree relatives (**figure 1B**) with a SQTS and BrS phenotype as described previously ^14^. To check whether the patient display BrS features, an ajmaline test was performed. After injection of ajmaline (1 mg/kg), the ECG recording showed a BrS type 1 ECG as published previously. Since the predominant phenotype (constant QTc=330 ms and aborted SCD) in the patient was SQTS, the study mainly focused on SQT5 and cells from the patient were named in the study as SQT5-cells or SQT5-hiPSC-CMs.

**Characterization of patient specific hiPSCs and hiPSC-CMs**

Beating cardiomyocytes were observed 8-12 days after starting the differentiation. HiPSC-CMs at age of day 40 to 60 were used for subsequent functional analyses. To examine the efficiency of differentiation, FACS (Fluorescence-activated Cell Sorting) analyses of differentiated cells stained with cardiac troponin T (cTnT) antibody were performed. More than 80%-90% of measured cells are cTnT-positive and α-actinin-positive, as shown in our previous studies^7, 8^. To check the maturity of hIPSC-CMs, immunostaining with antibodies against cardiac markers (a-actinin, cTnT and MLC2V) were performed to identify cardiomyocytes (**figure S1 F**). In addition, action potentials of cardiac morphology (**figure S2**) and sodium (SCN5A) and L-type calcium channel expression and currents, which are main functional features of cardiomyocytes, were measured. All the data showed that the hiPSC-CMs used possess similarity to human cardiomyocytes although some immature features like spontaneous beating exist.

**Peak I_Na_ was reduced in hiPSC-CMs from the patient**

The voltage-dependent activation was suppressed (the voltage at 50% activation was shifted to a more positive potential), however the inactivation and recovery from inactivation of I_Na_ did not differ significantly. The gene correction rescued I_Na_ (**figure 3_,_ S5 A-F**).

**Quinidine and amiodarone prolonged action potential duration (APD) in SQT5-hiPSC-CMs**

To investigate the responses of the SQT5-hiPSC-CMs to different antiarrhythmic drugs, we tested the APD-prolonging effects of quinidine, sotalol and amiodarone, which have been tested for treatment of SQTS, mainly for SQT1-patients. As expected, quinidine prolonged APD significantly (**figure S8 A-C**). Amiodarone showed effects similar to that of quinidine (**figure S9 A-C**). Surprisingly, sotalol failed to prolong APD and even slightly shortened APD at high concentration (**figure S10 A-C**). Considering that sotalol is a typical APD-prolonging drug, we repeated the measurements in hiPSC-CMs from the healthy donor. Indeed, sotalol prolonged APD in healthy cells (**figure S11**).

**Amiodarone attenuated occurrence of arrhythmic events in SQT5-hiPSC-CMs**

The APD-prolonging effects of quinidine and amiodarone suggest their possible antiarrhythmic effects in SQT5 cardiomyocytes. Therefore, their effects were examined in spontaneously beating SQT5-hiPSC-CMs showing arrhythmic episodes monitored by spontaneous calcium transients. Quinidine showed only a slight effect on the occurrence of arrhythmias. In 11 cells showing arrhythmic events, quinidine terminated arrhythmic events in only 3 cells (**figure S8D-E**). Sotalol showed almost no effect on arrhythmic events (**figure S10 D-E**). Amiodarone, however, significantly reduced the occurrence of arrhythmic events. In 6 out of 8 cells amiodarone terminated arrhythmic events (**figure S9 D-E**).

**Discussion**

With respect to cellular studies on BrS or SQTS associated with calcium channels, previous studies mainly used heterologous expression systems.

[Cordeiro](https://pubmed.ncbi.nlm.nih.gov/?term=Cordeiro+JM&cauthor_id=19358333) et al. ^9^expressed WT or T11I CACNB2b in TSA201 cells and found that both fast and slow decays of I(_Ca_) were enhanced in mutant channels between 0 and + 20 mV. They reported the first BrS mutation in CaCNB2b resulting in accelerated inactivation of L-type calcium channel current. Their results suggest that the faster current decay results in a loss-of-function responsible for the BrS phenotype.

[Burashnikov](https://pubmed.ncbi.nlm.nih.gov/?term=Burashnikov+E&cauthor_id=20817017) et al. ^10^reported that functional expression of two CACNA1C mutations associated with BrS and BrS+SQT led to loss of function in calcium channel current. This study for the first time identified CACNA2D1 as a novel BrS susceptibility gene and CACNA1C, CACNB2, and CACNA2D1 as possible novel early repolarization syndrome (ERS) susceptibility genes.

[Nakajima](https://pubmed.ncbi.nlm.nih.gov/?term=Nakajima+T&cauthor_id=22987075) et al ^11^ performed genetic analysis of BrS-causing genes including SCN5A, SCN1B, SCN3B, CACNA1C, CACNB2, KCNE3 and KCNE5 in 40 Japanese patients with Brugada-pattern electrocardiogram (ECG). Besides identifying 8 SCN5A mutations in the cohort, they found a KCNE3 T4A mutation in a 55-year-old male patient who had experienced several episodes of syncope. To characterize the functional consequence of the mutant, electrophysiological experiments using whole-cell patch-clamp methods and computer simulations using human right ventricular wall model were carried out. They found that KCNE3 T4A increased I_to_, which was recapitulated by heterologously coexpressing Kv4.3+KChIP2b+KCNE3-wild type or KCNE3-T4A in CHO cells, suggesting a gain of function of I_to_, which could underlie the pathogenesis of Brugada-pattern ECG. The data provide novel insights into the genetic basis of Japanese BrS.

In all aforementioned studies regarding BrS and calcium gene, mutations were investigated by sequencing and functional studies were carried out in heterologous expression systems, which are different from our system (hiPSC-CMs). All the studies connected mutations with clinical phenotypic features by changes of ion channel currents observed in heterologous expression systems. The heterologous expression cells differ from cardiomyocytes in many aspects, especially the cardiac specific electrical activity and cellular molecules that are important for the cardiac functions. Even though functional changes of ion channels expressed in heterologous expression systems can be detected, it is still not known whether the changes are enough for causing the clinical phenotypic changes like APD (representing QT interval) changes and arrhythmias. However, the hiPSC-CMs can show APD changes and arrhythmic events besides changes of ion channel currents. Therefore, hiPSC-CMs are more efficient than heterologous expression systems for modeling BrS and SQTS. In the present study it was able to use this model for studying the effect of different drugs and/or testing the role of PI3K pathway, which has not been studied using published models in the past yet.

It has been demonstrated that some genetic mutations can cause the overlapping syndromes in a single patient^12^. It is also known that BrS can be overlapped with SSS (sick sinus syndrome), CCD (cardiac conduction defect), AS (atrial standstill), and AF (atrial fibrillation) ^12, 13^. Those overlap-syndromes were found to be caused sodium channel mutations.

[Antzelevitch](https://pubmed.ncbi.nlm.nih.gov/?term=Antzelevitch+C&cauthor_id=17224476) et al.^14^ **reported that mutations in L-type calcium channel genes were associated with BrS overlapped with SQTS. In their study, g**enetic and heterologous expression studies revealed loss-of-function missense mutations in CACNA1C (A39V and G490R) and CACNB2 (S481L) encoding the alpha1- and beta2b-subunits of the L-type calcium channel. This was the first report of loss-of-function^6^ mutations in genes encoding the cardiac L-type calcium channel to be associated with a familial sudden cardiac death syndrome in which a Brugada syndrome phenotype is combined with short QT intervals.

In study of Antzelevitch et al. results suggested the association of mutations with the clinical phenotype of overlap syndrome (BrS+SQTS), but the genotype-phenotype correlation was not proved. In our study, the correlation was proved by three bodies of evidences: 1) diseased cells showed phenotypic changes comparing with healthy cells; 2) correction of the variant rescued the phenotypic changes; 3) insertion of the variant into wild-type cells recapitulated the phenotype. Our study demonstrated quite clearly that the variant (S480L) in CACNB2 gene is pathogenic in this patient with SQTS5 overlapped with BrS.

**Figure legends**

**Figure S1. Genetic correction of SQT5hiPSCs by CRISPR/Cas9 genome editing.** (A) Corrected hiPSCs were generated with a CRISPR guideRNA targeting the CACNB2 exon 20 and a single-stranded oligonucleotide (ssODN) for homology-directed repair. (B) Confirmation of genetic correction, assessed by Sanger sequencing of genomic DNA. Silent SNP was introduced by ssODN for PAM site mutation (asterisks). (C) Purity of patient-specific and CRISPR-corrected hiPSC lines was evaluated by flow cytometry analysis of pluripotency markers OCT4 and TRA1-60. Gray dots represent the negative controls. (D) Patients’ and CRISPR-corrected hiPSC lines exhibited a typical human stem cell-like morphology. Scale bar: 100 μm. (E) Immunofluorescence staining for key pluripotency markers OCT4, NANOG and TRA1-60 in patients’ and corrected hiPSC lines. Nuclei were counter-stained with DAPI. Scale bar: 100 μm.

**Figure S2. Action potentials recorded in hiPSC-CMs.** In all the three cell lines, most cells (around 76%) showed ventricular-like APs. APs with different forms were observed. Three types (ventricular-like, atrial-like and nodal-like) APs were distinguished by their resting potential, amplitude and the plateau phase as well as the ratio of APD90/APD50. Shown are representative traces of three forms of APs observed in SQT5-hiPSC-CMs.

**Figure S3. Action potential parameters in hiPSC-CMs.** Action potentials (AP) paced at 1 to 3 Hz were recorded and the AP parameters including the amplitude (APA) and resting potential (RP) of APs were compared among hiPSC-CMs from the SQT5-patient (SQT5), the healthy donor (Healthy) and CRISPR-corrected cells (Corrected). (A) Averaged values of APA in each cell line at 1 to 3 Hz. (B) Averaged values of RP in each cell line at 1 to 3 Hz. Spontaneous action potentials were recorded in spontaneously beating hiPSC-CMs from the patient (SQT5) and the healthy donor (Healthy). The action potential durations (APD50 and APD90) were corrected for beating frequency with Bazett´s correction. The corrected APDs (cAPD50 and cAPD90), the beating frequency and the maximum diastolic potential (MDP) were compared between the two groups. (C) Representative traces of spontaneous action potentials in a cell from the healthy donor (Healthy). (D) Representative traces of spontaneous action potentials in a cell from the patient (SQT5). (E) Averaged values of cAPD50 in SQT5 and healthy hiPSC-CMs. (F) Averaged values of cAPD90 in SQT5 and healthy hiPSC-CMs. (G) Averaged values of beating frequency in each cell line. (H) Averaged values of MDP in each cell line. The p-values versus Healthy were determined according to the analysis of t-test for two groups. “n.s.”, not significant.

**Figure S4. Protein level of CACNB2 decreased in SQT5-hiPSC-CMs.** Western blot and immunostaining analyses were performed to examine the expression level of CACNB2 and CACNA1C in hiPSC-CMs from the patient (SQT5), the healthy donor (Healthy) and the variant-corrected (Corrected) cells. (A) Representative examples of western blots of cell lysates from healthy donor cell line (Healthy), the patient (SQT5) and the CRISPR-corrected (Corrected) hiPSC-CMs. (B) Statistical analyses of relative expression level of CACNB2. (C) Statistical analyses of relative expression level of CACNA1C. Immunostaining analysis was performed to examine the expression level of CACNB2 and CACNA1C in cell membrane of hiPSC-CMs from the patient (SQT5), the healthy donor (Healthy) and the variant-corrected (Corrected) cells. (D) Representative examples of immunostaining with antibodies against CACNA1C and CACNB2. (E) Mean values of fluorescence intensity showing expression level of CACNA1C and CACNB2 in cell membrane. Data were shown as mean ± standard error of the mean (SEM). “n” numbers represent the number of experiments (biological replicates, B-C) or measured cells (E). *p<0.05 of SQT5 versus Healthy analyzed by one-way analysis of variance(ANOVA) with Holm-Sidak post-test.

**Figure S5. Kinetics of I_Na_.** (A) The activation curves of I_Na_ in cells from each group. (B) The half-maximum activation potential (V0.5) of I_Na_ in cells from each group. (C) The inactivation curves of I_Na_ in cells from each group. (D) The half-maximum inactivation potential (V0.5) of I_Na_ in cells from each group. (E) The curves of recovery from inactivation of I_Na_ in cells from each group. (F) The time constants (Tau) of recovery from inactivation of I_Na_ in cells from each group. *p<0.05 versus Healthy according to the analysis of one way ANOVA with Holm-Sidak post-test.

**Figure S6. Creation and characterization of S480L variant in a healthy hiPS cell line.** (A). Strategy of introducing S480L mutation. The PAM sequence of guide DNA (gDNA) was labeled with green colour, while two nucleotides to be changed by silent SNP in ssODN were labeled with red colour. (B) Kpn1 restriction digestion of PCR products from genomic DNA of GFP-positive iPSC clones. The positive clones indicated by boxes showed three bands (371,247 and 124bp respectively) after Kpn1 digestion. (C) Confirmation of S480L mutation generation in two representative clones by Sanger sequencing. Two silent SNPs introduced by ssODN for both Kpn1 digestion and to prevent repeat cutting were indicated by asterisks. (D) Representative karyotype of created clone#1 hiPSC line. (E) Representative Immunofluorescence staining image for key pluripotency markers OCT4 (red), NANOG (red), SOX2 (red) and SSEA4 (red) in created clone #1 hiPSC line. Nuclei were counter-stained with DAPI (blue). Scale bar: 50 μm.

**Figure S7. An introduced variant suppressed L-type calcium channel currents and changed action potentials.** The identical CACNB2 variant (c.1439C>T/p.S480L) was introduced in a healthy hiPSC line. Both the healthy and gene-edited hiPSCs were differentiated into cardiomyocytes and the action potential duration (APD) and L-type calcium channel currents (I_Ca-L_) were analyzed. (A) Representative action potential traces of healthy (Healthy) and gene-edited (Introduced) hiPSC-CMs at 1 Hz. (B) Averaged values of APD50 in each cell line at 1 Hz. (C) Averaged values of APD90 in each cell line at 1 Hz. (D) Mean values of maximal depolarization velocity (Vmax) of action potentials. (E) Representative traces of I_Ca-L_ in healthy hiPSC-CMs. (F) Representative traces of I_Ca-L_in gene-edited hiPSC-CMs. (G) The current-voltage (I-V) relationship curves of L-type calcium channel currents (I_Ca-L_) in cells from each group. (H) The activation curves of I_Ca-L_ in cells from each group. (I) The half-maximum activation potential (V0.5) of I_Ca-L_ in cells from each group. (J) The inactivation curves of I_Ca-L_ in cells from each group. (K) The half-maximum inactivation potential (V0.5) of I_Ca-L_ in cells from each group. (L) The curves of recovery from inactivation of I_Ca-L_ in cells from each group. (M) The time constants (Tau) of recovery from inactivation of I_Ca-L_ in cells from each group. “n” numbers represent the number of cells. *p<0.05 versus Healthy according to the analysis of t-test for two groups.

**Figure S8. Quinidine prolonged action potential duration but failed to suppress arrhythmic event in SQT5-hiPSC-CMs.** Action potentials (APs) paced by 1 to 3 Hz stimulations and spontaneous calcium transients were recorded in SQT5-hiPSC-CMs in absence and presence of 10 µM quinidine. The duration of APs at 50% (APD50) and 90% (APD90) repolarization were analyzed (n=12). (A) Representative action potential traces in absence (Ctr) and presence of 10 µM quinidine (Qui) at 1 Hz. (B) Averaged values of APD50 in absence (Ctr) and presence of 10 µM quinidine at 1 to 3 Hz. (C) Averaged values of APD90 in absence (Ctr) and presence of 10 µM quinidine at 1 to 3 Hz. (D) Representative traces of calcium transients in a SQT5-hiPSC-CM in absence (Ctr) and presence of quinidine. (E) Percentage of cells showing arrhythmic events in absence (Ctr) and presence of quinidine. “n” represents the number of cells. *p<0.05 versus Ctr, according to the analysis of paired t-test.

**Figure S9. Amiodarone prolonged action potential duration and suppressed arrhythmic event in SQT5-hiPSC-CMs.** Action potentials (APs) paced by 1 to 3 Hz stimulations and spontaneous calcium transients were recorded in SQT5-hiPSC-CMs in absence and presence of 0.1, 1 and 10 µM amiodarone. The duration of APs at 50% (APD50) and 90% (APD90) repolarization were analyzed (n=13). (A) Representative action potential traces in absence (Ctr) and presence of 0.1 µM amiodarone at 1 Hz. (B) Averaged values of APD50 in absence (Ctr) and presence of 0.1, 1 and 10 µM quinidine at 1 to 3 Hz. (C) Averaged values of APD90 in absence (Ctr) and presence of 0.1, 1 and 10 µM quinidine at 1 to 3 Hz. (D) Representative traces of calcium transients in a SQT5-hiPSC-CM in absence (Ctr) and presence of 0.1 µM amiodarone. (E) Percentage of cells showing arrhythmic events in absence (Ctr) and presence of 0.1 µM amiodarone. “n” represents the number of cells. *p<0.05 versus Ctr, according to the analysis of paired t-test.

**Figure S10. Sotalol failed to prolong action potential duration and suppress arrhythmic event in SQT5-hiPSC-CMs.** Action potentials (APs) paced by 1 to 3 Hz stimulations and spontaneous calcium transients were recorded in SQT5-hiPSC-CMs in absence and presence of 10, 30 and 100 µM sotalol. The duration of APs at 50% (APD50) and 90% (APD90) repolarization were analyzed (n=9). (A) Representative action potential traces in absence (Ctr) and presence of 10 µM sotalol at 1 Hz. (B) Averaged values of APD50 in absence (Ctr) and presence of 10, 30 and 100 µM sotalol at 1 to 3 Hz. (C) Averaged values of APD90 in absence (Ctr) and presence of 10, 30 and 100 µM sotalol at 1 to 3 Hz. (D) Representative traces of calcium transients in a SQT5-hiPSC-CM in absence (Ctr) and presence of sotalol. (E) Percentage of cells showing arrhythmic events in absence (Ctr) and presence of sotalol. “n” represents the number of cells.

**Figure S11. Sotalol prolonged action potential duration of healthy hiPSC-CMs.** Action potentials (APs) paced by 1 Hz stimulations were recorded in healthy hiPSC-CMs in absence and presence of 30 µM sotalol. The duration of APs at 50% (APD50) and 90% (APD90) were analyzed. (A) Representative action potential traces in absence (Ctr) and presence of 30 µM sotalol. (B) Averaged values of APD50 in absence (Ctr) and presence of 30 µM sotalol. (C) Averaged values of APD90 in absence (Ctr) and presence of 30 µM sotalol. “n” represents the number of cells. *p<0.05 versus Ctr according to the analysis of paired t-test.

**References**

1. El-Battrawy I, Lan H, Cyganek L, Zhao Z, Li X, Buljubasic F, Lang S, Yucel G, Sattler K, Zimmermann WH, Utikal J, Wieland T, Ravens U, Borggrefe M, Zhou XB and Akin I. Modeling Short QT Syndrome Using Human-Induced Pluripotent Stem Cell-Derived Cardiomyocytes. *J Am Heart Assoc*. 2018;7.

2. Garg P, Oikonomopoulos A, Chen H, Li Y, Lam CK, Sallam K, Perez M, Lux RL, Sanguinetti MC and Wu JC. Genome Editing of Induced Pluripotent Stem Cells to Decipher Cardiac Channelopathy Variant. *J Am Coll Cardiol*. 2018;72:62-75.

3. Itzhaki I, Maizels L, Huber I, Zwi-Dantsis L, Caspi O, Winterstern A, Feldman O, Gepstein A, Arbel G, Hammerman H, Boulos M and Gepstein L. Modelling the long QT syndrome with induced pluripotent stem cells. *Nature*. 2011;471:225-9.

4. Matsa E, Rajamohan D, Dick E, Young L, Mellor I, Staniforth A and Denning C. Drug evaluation in cardiomyocytes derived from human induced pluripotent stem cells carrying a long QT syndrome type 2 mutation. *Eur Heart J*. 2011;32:952-62.

5. Ballini M, Muller J, Livi P, Chen Y, Frey U, Stettler A, Shadmani A, Viswam V, Jones IL, Jackel D, Radivojevic M, Lewandowska MK, Gong W, Fiscella M, Bakkum DJ, Heer F and Hierlemann A. A 1024-Channel CMOS Microelectrode Array With 26,400 Electrodes for Recording and Stimulation of Electrogenic Cells In Vitro. *IEEE J Solid-State Circuits*. 2014;49:2705-2719.

6. Campuzano O, Fernandez-Falgueras A, Lemus X, Sarquella-Brugada G, Cesar S, Coll M, Mates J, Arbelo E, Jorda P, Perez-Serra A, Del Olmo B, Ferrer-Costa C, Iglesias A, Fiol V, Puigmule M, Lopez L, Pico F, Brugada J and Brugada R. Short QT Syndrome: A Comprehensive Genetic Interpretation and Clinical Translation of Rare Variants. *J Clin Med*. 2019;8.

7. Huang M, El-Battrawy I, Zhou X and Akin I. alpha1-adrenoceptors and takotsubo syndrome: pathophysiologic connotations-Authors' reply. *Europace*. 2021;23:1152.

8. Hanses U, Kleinsorge M, Roos L, Yigit G, Li Y, Barbarics B, El-Battrawy I, Lan H, Tiburcy M, Hindmarsh R, Lenz C, Salinas G, Diecke S, Muller C, Adham I, Altmuller J, Nurnberg P, Paul T, Zimmermann WH, Hasenfuss G, Wollnik B and Cyganek L. Intronic CRISPR Repair in a Preclinical Model of Noonan Syndrome-Associated Cardiomyopathy. *Circulation*. 2020;142:1059-1076.

9. Cordeiro JM, Marieb M, Pfeiffer R, Calloe K, Burashnikov E and Antzelevitch C. Accelerated inactivation of the L-type calcium current due to a mutation in CACNB2b underlies Brugada syndrome. *J Mol Cell Cardiol*. 2009;46:695-703.

10. Burashnikov E, Pfeiffer R, Barajas-Martinez H, Delpon E, Hu D, Desai M, Borggrefe M, Haissaguerre M, Kanter R, Pollevick GD, Guerchicoff A, Laino R, Marieb M, Nademanee K, Nam GB, Robles R, Schimpf R, Stapleton DD, Viskin S, Winters S, Wolpert C, Zimmern S, Veltmann C and Antzelevitch C. Mutations in the cardiac L-type calcium channel associated with inherited J-wave syndromes and sudden cardiac death. *Heart Rhythm*. 2010;7:1872-82.

11. .

12. Makita N. Phenotypic overlap of cardiac sodium channelopathies: individual-specific or mutation-specific? *Circ J*. 2009;73:810-7.

13. Remme CA, Wilde AA and Bezzina CR. Cardiac sodium channel overlap syndromes: different faces of SCN5A mutations. *Trends Cardiovasc Med*. 2008;18:78-87.

14. Antzelevitch C, Pollevick GD, Cordeiro JM, Casis O, Sanguinetti MC, Aizawa Y, Guerchicoff A, Pfeiffer R, Oliva A, Wollnik B, Gelber P, Bonaros EP, Jr., Burashnikov E, Wu Y, Sargent JD, Schickel S, Oberheiden R, Bhatia A, Hsu LF, Haissaguerre M, Schimpf R, Borggrefe M and Wolpert C. Loss-of-function mutations in the cardiac calcium channel underlie a new clinical entity characterized by ST-segment elevation, short QT intervals, and sudden cardiac death. *Circulation*. 2007;115:442-9.
