# Supplementary figures and images for: Deciphering the pathogenic role of a variant with uncertain significance for short QT and Brugada syndromes using gene‐edited human‐induced pluripotent stem cell‐derived cardiomyocytes and preclinical drug screening
Source: Clin Transl Med. 2021 Dec 26;11(12):e646. doi: 10.1002/ctm2.646 (PMC8710296; doi:10.1002/ctm2.646)

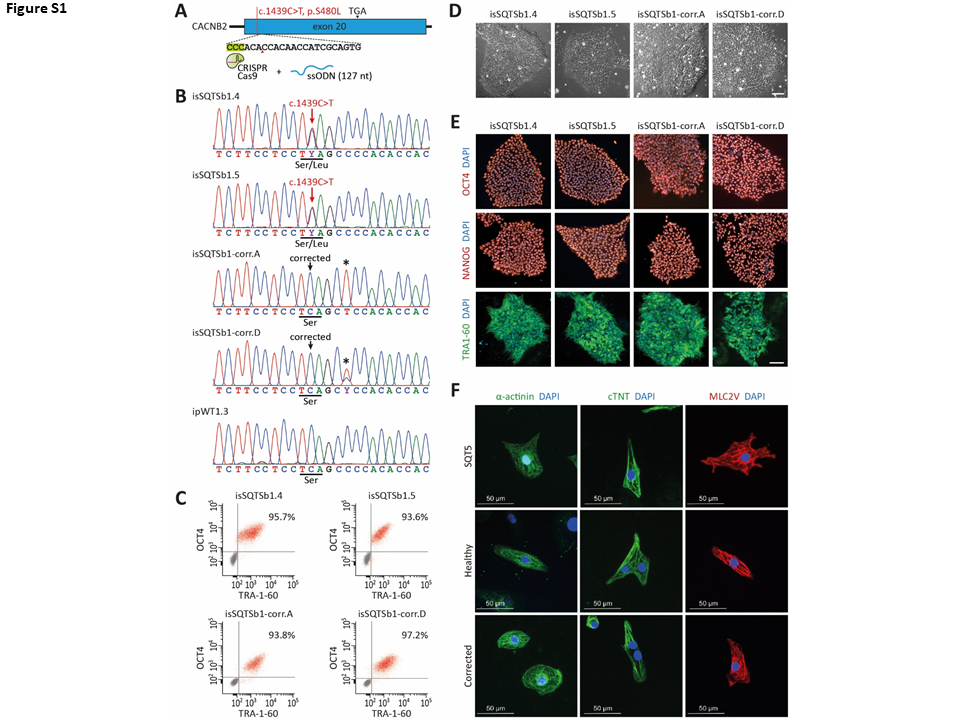

Supplement: Supplementary file 2 — Supporting Information‐Supplementary figure 1 [file CTM2-11-e646-s007.TIF]

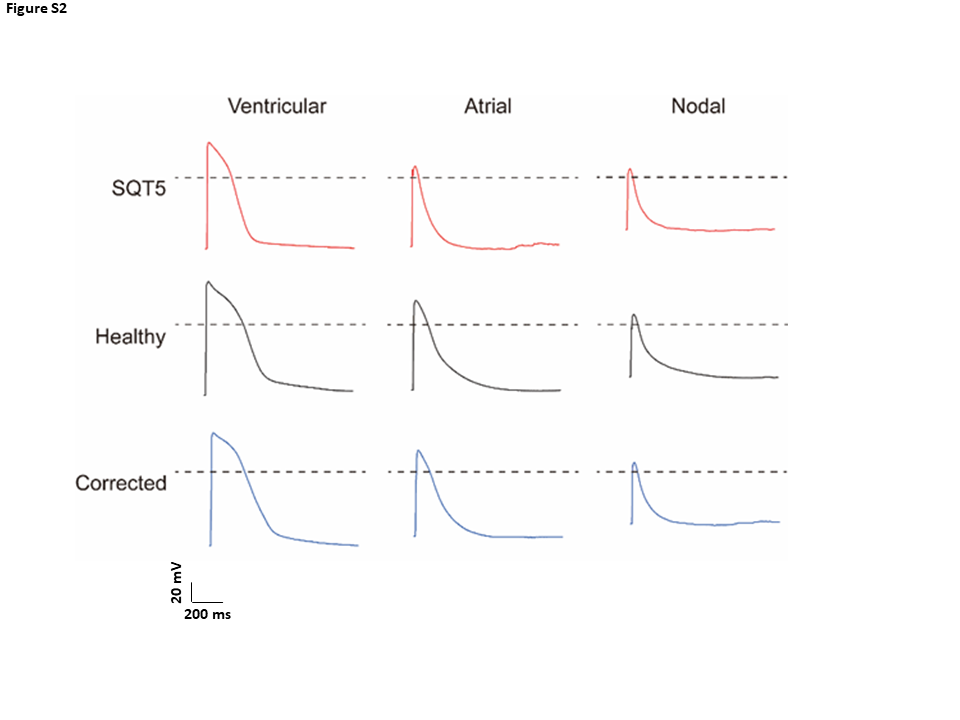

Supplement: Supplementary file 3 — Supporting Information‐Supplementary figure 2 [file CTM2-11-e646-s004.TIF]

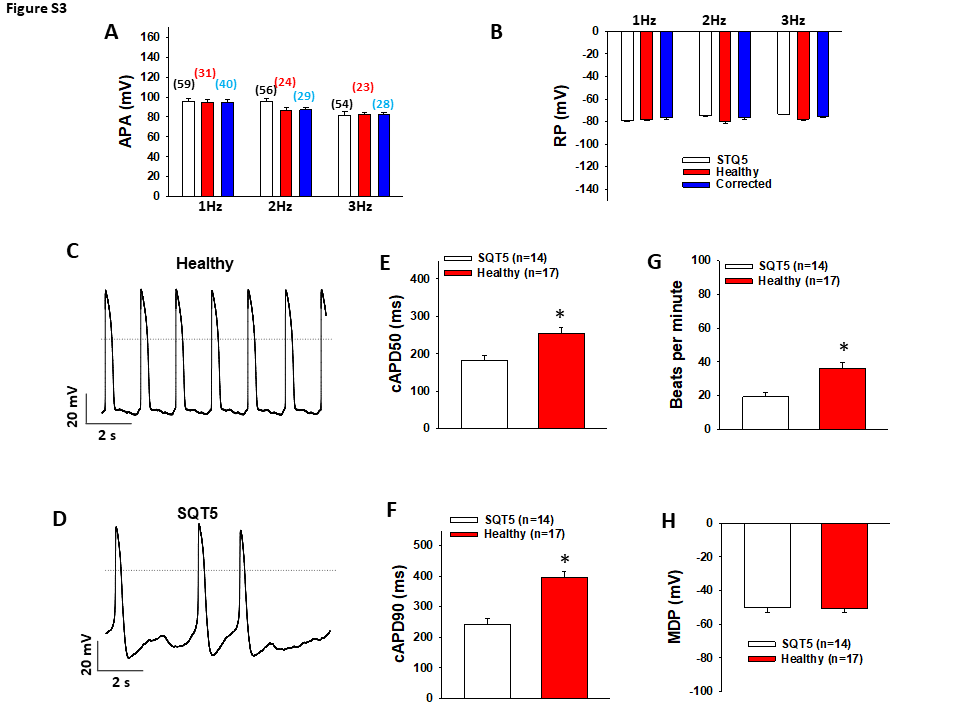

Supplement: Supplementary file 4 — Supporting Information‐Supplementary figure 3 [file CTM2-11-e646-s002.TIF]

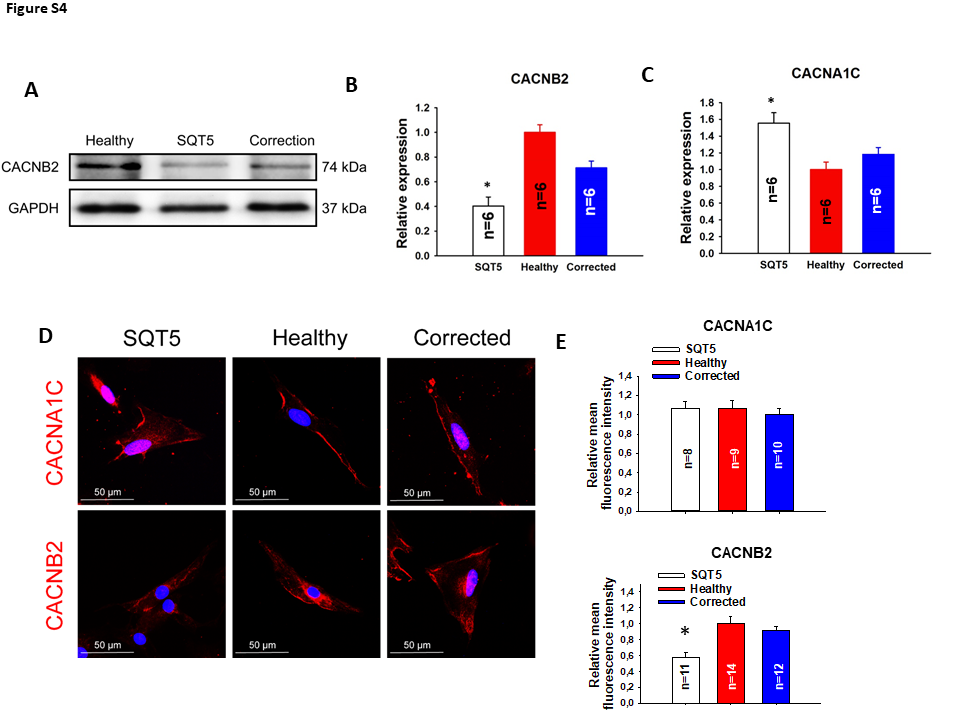

Supplement: Supplementary file 5 — Supporting Information‐Supplementary figure 4 [file CTM2-11-e646-s001.TIF]

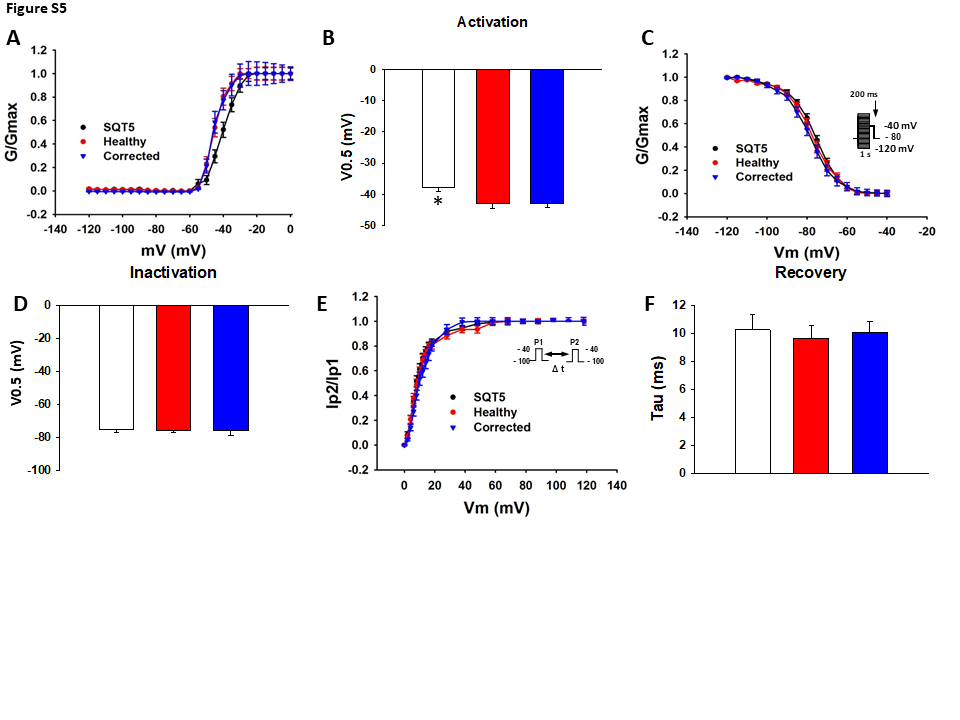

Supplement: Supplementary file 6 — Supporting Information‐Supplementary figure 5 [file CTM2-11-e646-s003.TIF]

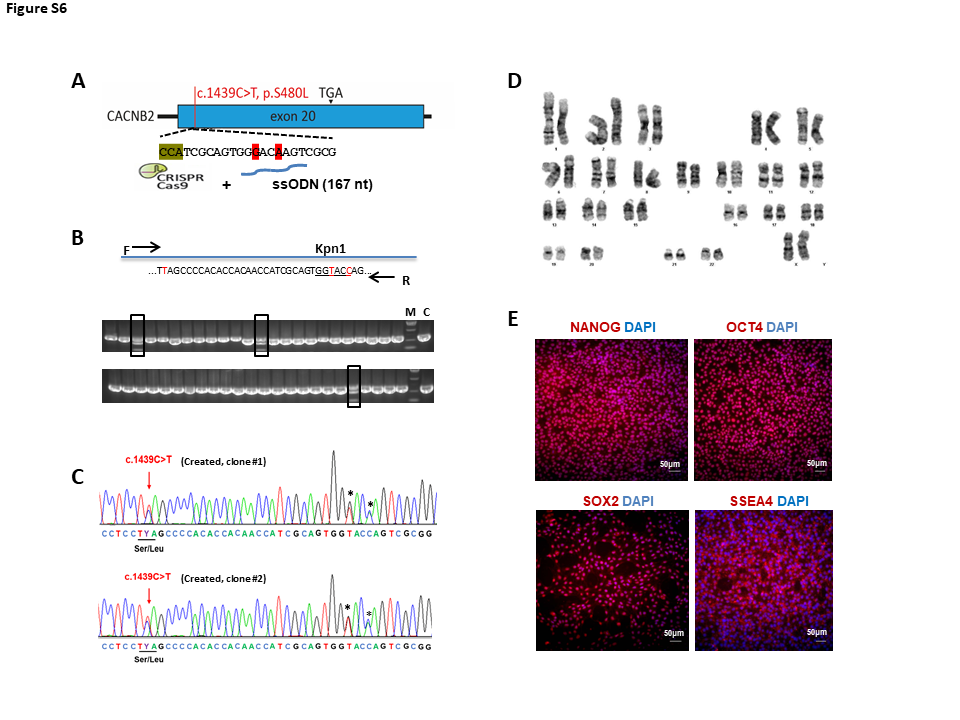

Supplement: Supplementary file 7 — Supporting Information‐Supplementary figure 6 [file CTM2-11-e646-s012.TIF]

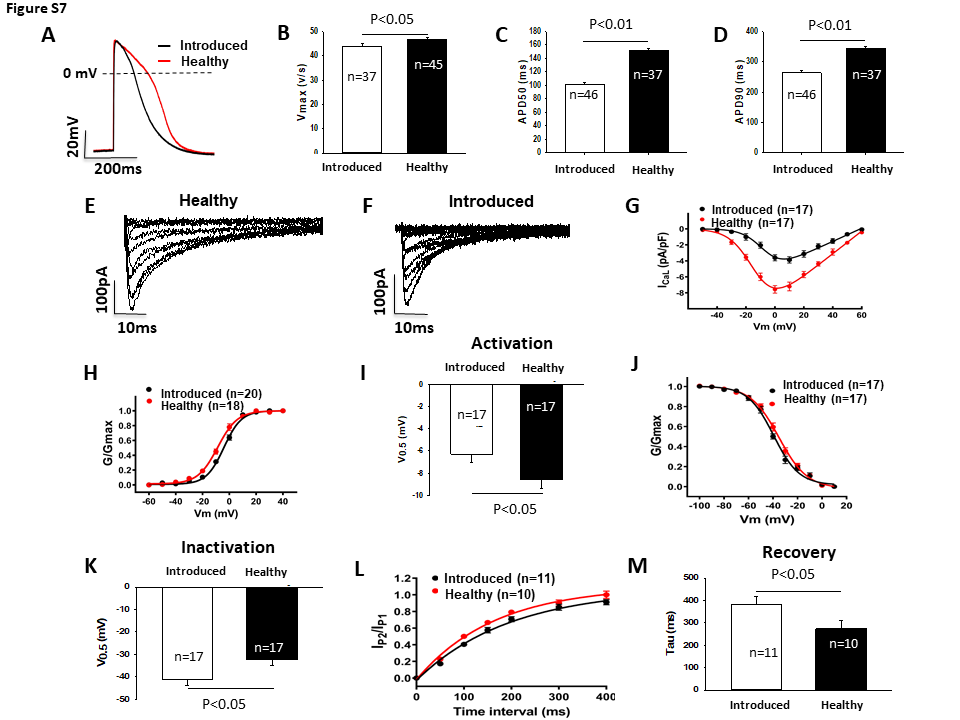

Supplement: Supplementary file 8 — Supporting Information‐Supplementary figure 7 [file CTM2-11-e646-s011.TIF]

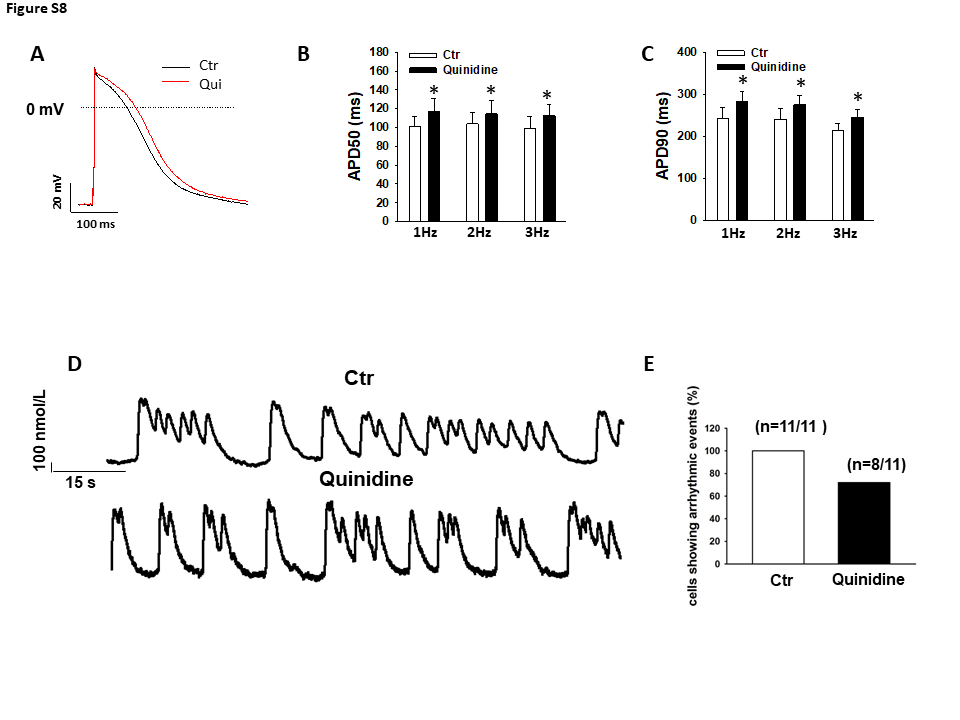

Supplement: Supplementary file 9 — Supporting Information‐Supplementary figure 8 [file CTM2-11-e646-s010.TIF]

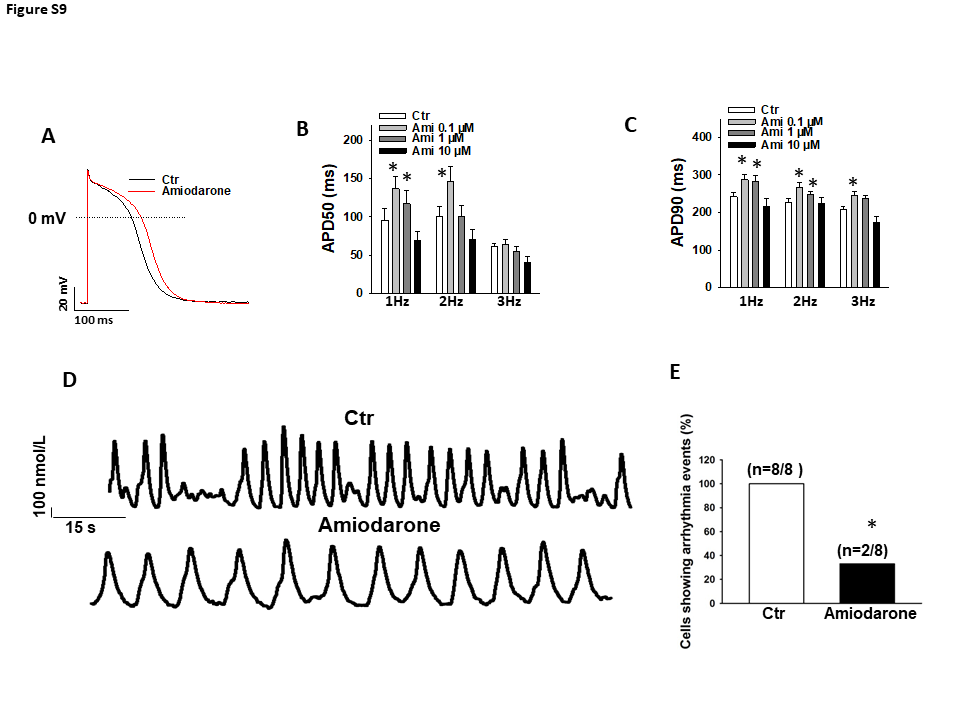

Supplement: Supplementary file 10 — Supporting Information‐Supplementary figure 9 [file CTM2-11-e646-s009.TIF]

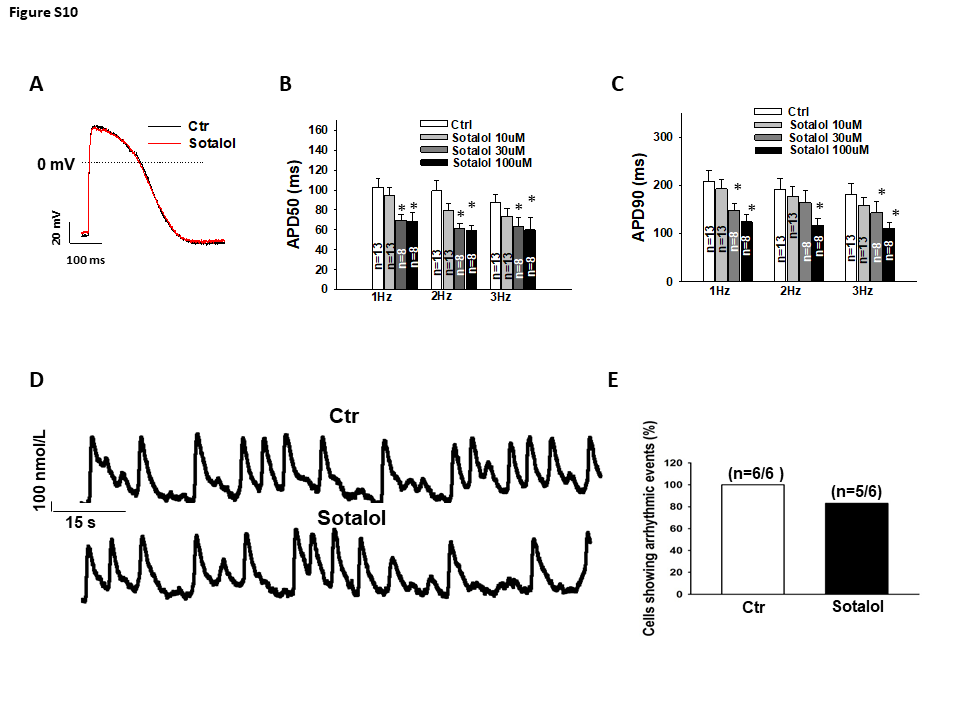

Supplement: Supplementary file 11 — Supporting Information‐Supplementary figure 10 [file CTM2-11-e646-s005.TIF]

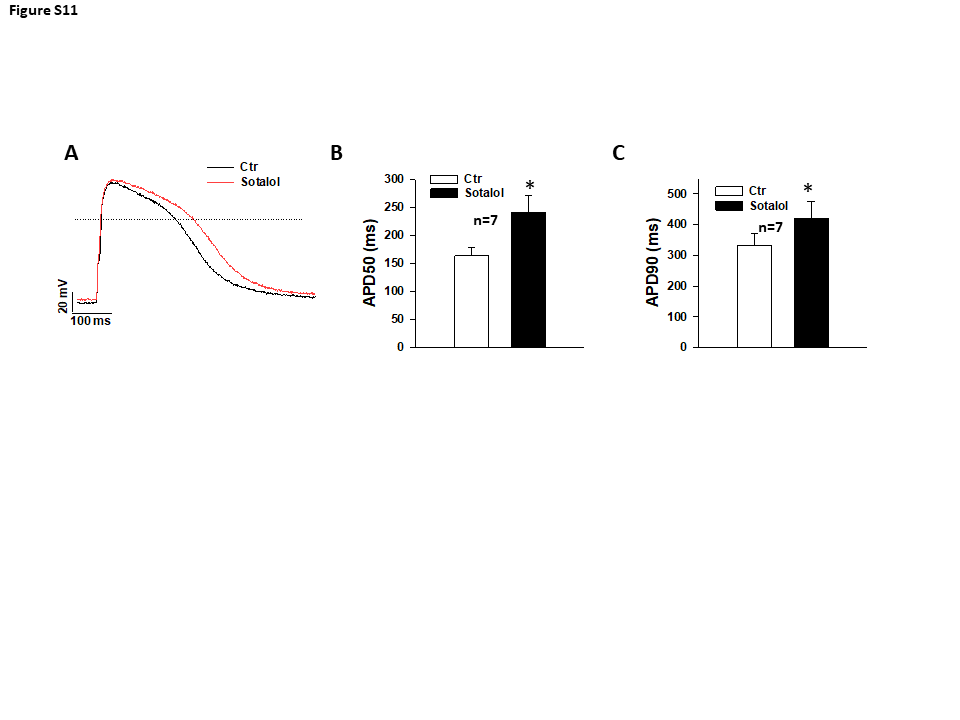

Supplement: Supplementary file 12 — Supporting Information‐Supplementary figure 11 [file CTM2-11-e646-s008.TIF]
